# A miniaturized culture platform for control of the metabolic environment

**DOI:** 10.1063/5.0169143

**Published:** 2024-03-01

**Authors:** Marta K. Orlowska, James R. Krycer, Janice D. Reid, Richard J. Mills, Michael R. Doran, James E. Hudson

**Affiliations:** 1 Cardiac Bioengineering Laboratory, QIMR Berghofer Medical Research Institute, Brisbane, Queensland, Australia; 2 School of Biomedical Sciences, Faculty of Health, Queensland University of Technology, Brisbane, Queensland, Australia; 3 School of Biomedical Sciences, Faculty of Medicine, University of Queensland, Brisbane, Queensland, Australia; 4 The Novo Nordisk Foundation Center for Stem Cell Medicine, reNEW, Murdoch Children's Research Institute, Melbourne, Australia; 5 Translational Research Institute, Woolloongabba, Brisbane, Queensland, Australia

## Abstract

The heart is a metabolic “omnivore” and adjusts its energy source depending on the circulating metabolites. Human cardiac organoids, a three-dimensional *in vitro* model of the heart wall, are a useful tool to study cardiac physiology and pathology. However, cardiac tissue naturally experiences shear stress and nutrient fluctuations via blood flow *in vivo*, whilst *in vitro* models are conventionally cultivated in a static medium. This necessitates the regular refreshing of culture media, which creates acute cellular disturbances and large metabolic fluxes. To culture human cardiac organoids in a more physiological manner, we have developed a perfused bioreactor for cultures in a 96-well plate format. The designed bioreactor is easy to fabricate using a common culture plate and a 3D printer. Its open system allows for the use of traditional molecular biology techniques, prevents flow blockage issues, and provides easy access for sampling and cell assays. We hypothesized that a perfused culture would create more stable environment improving cardiac function and maturation. We found that lactate is rapidly produced by human cardiac organoids, resulting in large fluctuations in this metabolite under static culture. Despite this, neither medium perfusion in bioreactor culture nor lactate supplementation improved cardiac function or maturation. In fact, RNA sequencing revealed little change across the transcriptome. This demonstrates that cardiac organoids are robust in response to fluctuating environmental conditions under normal physiological conditions. Together, we provide a framework for establishing an easily accessible perfusion system that can be adapted to a range of miniaturized cell culture systems.

## I. INTRODUCTION

**FIG. 1.**
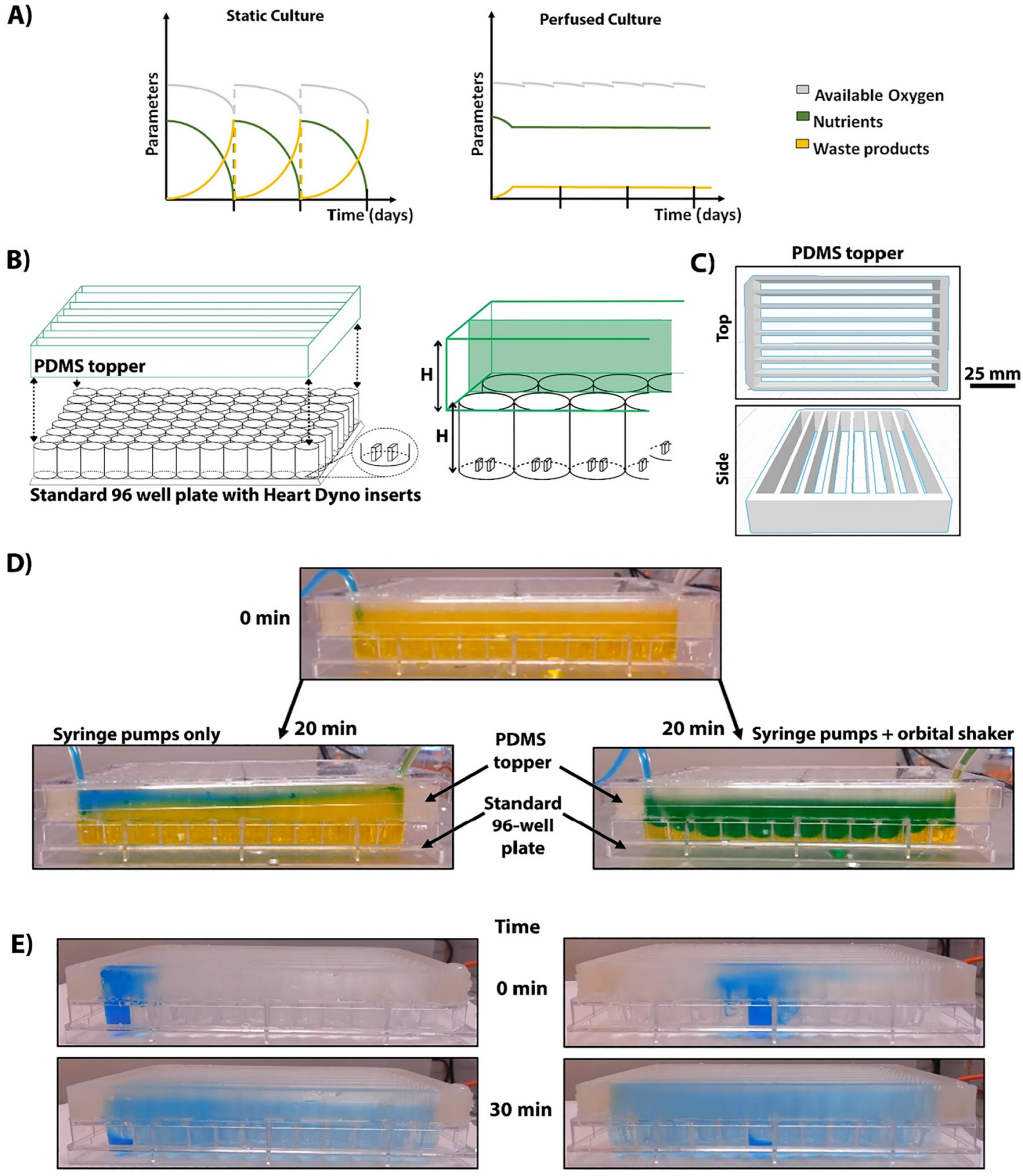
“Proof of concept” bioreactor design indicates the need for mixing motion together with perfusion for proper medium propagation. (a) Schematics showing the available dissolved oxygen (gray), consumption of nutrients (green), and buildup of waste products (yellow) between static and perfused (bioreactor) cultures across days. All parameters oscillate in static culture due to medium changes, whereas in perfused culture, nutrients and waste products stay constant across culturing time. (b) Preliminary bioreactor design utilizing a PDMS-cast topper glued using uncured PDMS on top of the standard 96-well plate, containing individually punched and glued Heart-Dyno inserts. H represents the height of the standard well, which is also the height used for the PDMS topper. (c) A rendered schematic of the cast PDMS topper presented from atop and side. Topper consists of eight channels allowing free flow between 12 wells. (d) New (blue) and residual (yellow) dye imitating medium kinetics using a syringe pump movement alone (top), the image depicts 20 min after perfusion at 5 ml/h, and with shaking (bottom) at 60 rpm, the image taken 20 min after mixing start. Fluid behavior did not change past 20 min time point. (e) Dye movement within the bioreactor relying on the orbital shaker alone. The height of standard wells prevented the equilibration of the dye between all wells. There is visible residual blue dye in the wells even after 30 min. Presented images are of propagation of the dye starting from the first or sixth well.

Modern cell culture methods aim to recreate the complex physical and physiochemical environment found in tissues. This is conventionally performed with static cultures, where the culture medium is manually exchanged (“refreshed”) at regular intervals. Between medium exchanges, nutrient concentrations decline while waste product concentrations increase and this relationship is inverted when the spent medium is refreshed [Fig. 1(a)]. Therefore, medium refreshment provides necessary new nutrients to support proper cellular development as well as removal of by-products produced by the cells. Some of those by-products can be toxic waste, others soluble factors and extracellular vesicles, together constituting the cellular secretome necessary for cellular communication and creation of favorable environmental niche, which needs restoring with each medium change. We have found that this causes acute, widespread changes across the metabolome in adipocytes^1^ and has functional implications on cardiomyocytes (CMs),^2,3^ which, in turn, may confound experiments. Therefore, a more physiological solution such as continuous medium perfusion may be necessary to maintain a more stable environment for the cells, through both the maintenance of nutrient levels and signaling molecules, as well as the removal of cellular by-products [Fig. 1(a)]. Furthermore, perfusion produces laminar or convective flow, which increases mass-transfer rates, and, thus, may reduce gradients that form in cell culture systems.^4^

The last few decades have seen a trend toward tissue culture in multi-well plates. This permitted a decrease in the medium volume and increased throughput to test multiple conditions at once, but its miniature format presents a challenge for perfusion systems. Others have engineered elegant perfusion culture devices based on 96-well plate formats.^5–7^ However, many of these technologies have sophisticated designs, which can be difficult to replicate or adopt.^5,7,8^ Additionally, many of these designs are incorporated into plate lids, which prevents easy access to the cells and sampling.^6,7^ Furthermore, systems relying on perfusion alone without stirring or mixing lead to slow equilibration and buildup of gradients between wells.^6^ Therefore, widespread adoption of multi-well perfusion systems could be facilitated by platforms that are easy to fabricate and use, and allow for non-invasive assaying and reduced heterogeneity in cell culture.

In this study, we developed a perfusion system that meets these criteria. With this platform, we tested the long-term impact of environmental stability on cellular metabolism and function. We focused on a model of cardiac tissue, using developed in our lab human cardiac organoids (hCOs), which consist of ∼70% CMs and ∼30% stromal cells^9^ representing major subgroups within the heart *in vivo*. As the heart naturally experiences shear stress associated with contractility^10^ and nutrient fluctuations through metabolic exchange between stromal cells in the interstitial spaces surrounding microvessels and CMs *in vivo*,^11–13^ we explored hCO behavior in a perfused environment. The already developed hCO platform is based on a 96-well plate format,^14^ enabling us to develop and test an inexpensive, simple perfusion bioreactor for miniaturized culture systems. Comparing this to conventional static culture, we found that although the perfusion system stabilized hCO metabolism, the contractile function and the transcriptome were relatively unchanged, thus demonstrating the robustness of cardiac tissue in response to environmental fluctuations under physiological conditions. In doing so, we have developed a perfusion system that can be applied to other cell-based models cultured in a microplate format.

## II. RESULTS

### A. Design and development of the bioreactor

To study the role of a stable cellular microenvironment on cardiac physiology, we decided to design a perfusion bioreactor compatible with our existing hCO platform. Our Heart-Dyno hCO platform^14^ consists of two elastomeric polydimethylsiloxane (PDMS) poles based at the bottom of the 96-well plate wells [Fig. 1(b)]. A pre-differentiated cell mixture of cardiac cells, is seeded around those poles, through self-assembly creating a functional hCO. The resistance from the poles allows for mechanical loading and proper structural organization, promotes the maturation of the hCO, and provides a means to assess their contractile function.

Our perfusion system needed to be optimized to facilitate (1) the quick establishment of medium equilibrium, (2) even media spread across all hCO, and (3) the removal of by-products gradually and consistently. This is important to establish a stable and homogenous cellular microenvironment between wells.

To empirically establish critical elements necessary for our bioreactor desired function, we initially designed the perfusion system by allowing the free flow of media between wells (“proof of concept”). This employs the easiest modification and a typical strategy when applying perfusion systems to multi-well plates. To achieve this, we first created a PDMS plate topper, a scaffold increasing the height of the outer walls of each row [Figs. 1(b) and 1(c)] without modification to the 96-well plate. The height of the topper was kept the same height as the wells themselves, allowing enough space for media to freely cross between 12 wells without splashing to neighboring corridors.

We assessed perfusion function using food dyes, which are of similar density to water, and at room temperature to avoid differences in the particle behavior. To emphasize the mixing and diffusion processes, two colored dyes were used, yellow dye to mark the original medium in the system and blue dye to mark the incoming, fresh medium. Obtaining green color from those two dyes would indicate homogenous mixing. The perfusion of this system solely via syringe pumps did not facilitate sufficient mixing between the perfused medium in the topper down to the bottom of the wells [Fig. 1(d)]. At slower flow rates, 1 and 5 ml/h [Fig. 1(d)] laminar flow was observed in the topper with limited diffusion into the wells. An enhanced flow rate of 90 ml/h showed medium propagation across all wells (∼10 min; data not shown). However, even though a faster flow is more physiologically relevant, we wanted to be able to run our system at a rate comparable to hCO medium exchange using our standard protocol. Considering the increase in the volume of media within the system from 150 *μ*l per well to ∼1 ml per well, additional mixing aids were required. Therefore, we placed the bioreactor on an orbital shaker, by testing a variety of the speeds we settled for 60 rpm, as speeds higher than 80 rpm caused the media to overflow and crossover into neighboring rows. The addition of rotating motion substantially improved medium mixing from well to well [Fig. 1(d)]. Nevertheless, the homogenous mixture after 20 min was only visible from the third to tenth well, making only 67% of the plate usable. We then proceeded to establish the speed at which the culture media becomes homogenous using movement from the orbital shaker alone. This time a single dye was used against water to provide better contrast for dye propagation. Dye was placed in the well, and the rest of the bioreactor was filled with water [Fig. 1(e)]. We tested dye dissemination from every well individually, each providing the same results. Spread of the dye across wells took over 30 min without homogeneity being established, as the residual dye remained at a higher concentration in the well it was originally placed in [Fig. 1(e)]. It led us to conclude that this system does not meet our requirements of quick and consistent establishment of equilibrium, likely due to insufficient mixing from the top-bottom of the wells.

**FIG. 2.**
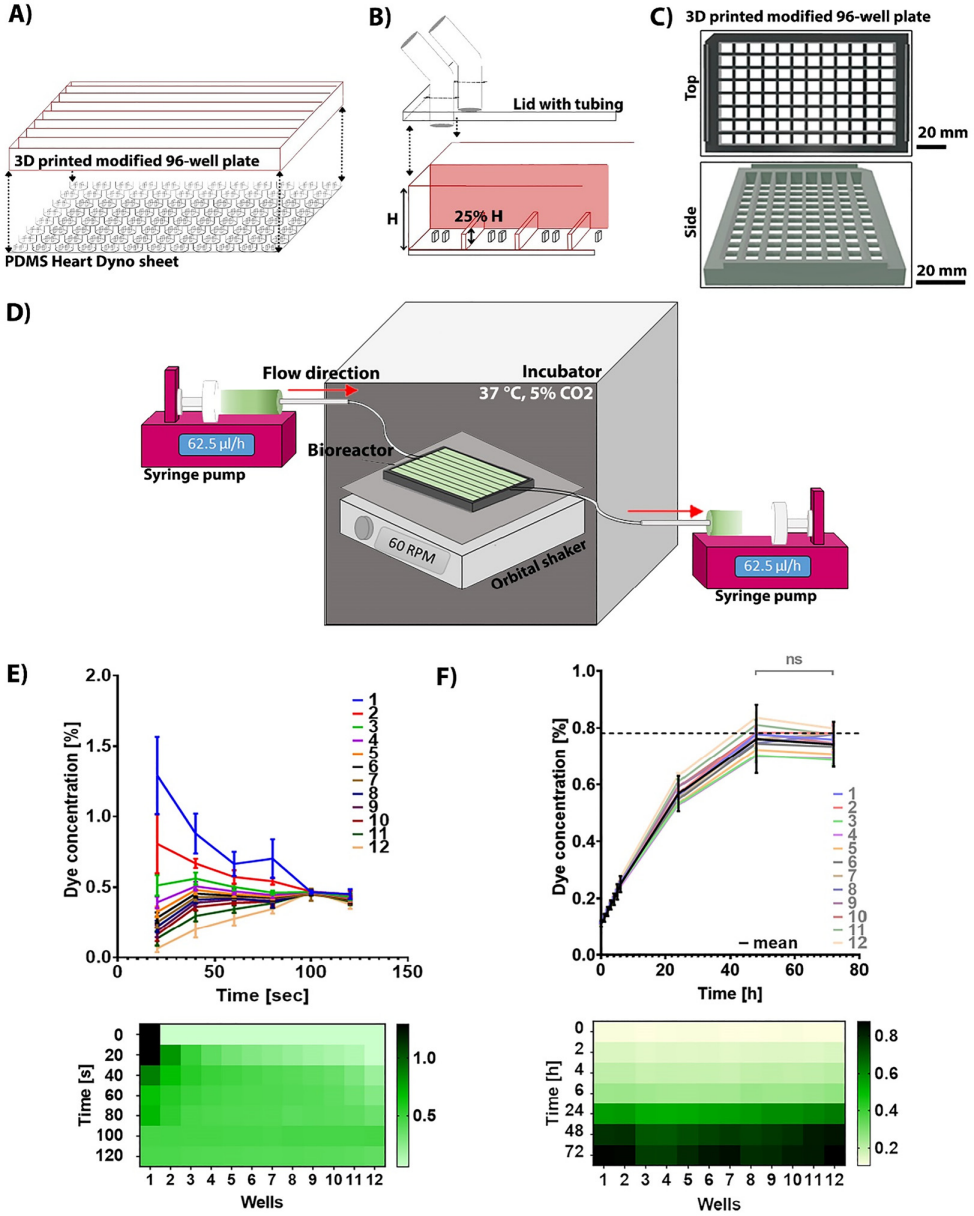
Optimal bioreactor design and function optimization. (a) Schematic of the final version of the bioreactor consisting of a 3D printed modified bottomless 96-well plate and PDMS Heart-Dyno sheet. (b) Modifications to the 96-well plate included the creation of eight corridors with 12 compartments each; between the compartments, the walls were reduced by 75%. Openings of the shape and dimensions of the outer tubing diameter were drilled in a lid to accommodate the tight fitting of tubing. (c) Schematic of the modified 96-well plate 3D printouts. (d) Diagram of the flow within the bioreactor once connected to syringe pumps and placed on an orbital shaker. The orbital shaker with the plate is placed within the controlled environment of an incubator, set at 5% CO_2_ and 37 °C. Syringe pumps together with media remain outside the incubator covered to prevent light exposure. (e) Mixing of fresh media reaches an equilibrium across all wells within 100 s, measured using a spectrophotometer. Heat map represents the mean dye intensity at different time-points across wells. n = 8 experimental runs. Data presented as mean with SD. (f) Using medium perfusion via syringe pumps, liquid is exchanged by the calculated residence time of 48 h. Dotted line marks the concentration of the input dye 0.78125%. Heat map represents the mean dye intensity at different time-points across wells. n = 4 experimental runs. Data presented as mean with SD. Two-way ANOVA with Sidak's post hoc multiple comparisons test was used to establish significance. p > 0.05 deemed insignificant.

Based on the “proof of concept” design findings, we redesigned the bioreactor to include the following (a summary can be found in Table 1 in the supplementary material):

• Each row of 12 wells was merged into a single corridor [Fig. 2(a)]. Well walls within each corridor were reduced to 25% of the well height to facilitate rapid top-bottom mixing [Fig. 2(b)]. By retaining 25% of the height, the working volume is still 110 *μ*l, allowing the maintenance of separate organoids in each well, and any shear stress created by fluid flow is reduced, limiting the number of hCO that could be pushed off the poles due to mechanical forces. Furthermore, the end wells remained empty to prevent impact caused by inlet/outlet tubing [Fig. 2(a)].
• Specific to our application, we designed the 96-well plate to be bottomless so we could attach a PDMS-cast Heart-Dyno platform as a bottom layer. By being able to attach the Heart-Dyno platform, the standard methodologies of seeding and culturing hCO could be maintained.^14^ Additionally, as PDMS is transparent, all our developed molecular and screening technologies could be used without modifications. Furthermore, circular wells were redesigned into squares to provide a larger surface area for the attachment of the PDMS layer and prevent bursting [Figs. 2(b) and 2(c)].
• Our new 96-well plate was 3D printed (design publicly available in a Tinkercad Gallery see Methods), creating a rapid and repeatable production of the plates [Fig. 2(c)].
• To provide tight inlets for the perfusion flow tubing, we drilled holes into the plate’s lid. These holes were the same size as the tubing's outer diameter and aligned at the center of the first and last wells of each corridor [Fig. 2(b)].
• To perfuse the bioreactor, syringe pumps were utilized to provide a consistent flow of the new media into the first well and uptake of the old media from the last well [Fig. 2(d)].
• To facilitate full mixing without regional differences, the bioreactor was placed on an orbital shaker (set to 60 rpm) within the CO_2_ incubator [Fig. 2(d)].

Due to the increase in the surface area in the bioreactor's wells, the volume per well was increased from 150 (the standard volume used in a 96-well plate) to 250 *μ*l. This enabled each well to be fully covered and the liquid interconnected between the wells.

We validated the medium propagation aided by the orbital shaker by measuring dye movement between wells [Fig. 2(e)]. It took 100 s for the dye to become homogenous across all wells, thus demonstrating quick medium equilibration.

Syringe pumps were then added to the apparatus, creating perfusion. The flow rate was calculated to equal the medium exchange rate in a standard culture, every 48 h, enabling direct comparison to standard culture. In our bioreactor, 12 wells are connected, resulting in a volume of 3 ml per corridor, requiring a flow rate of 62.5 *μ*l/h per corridor. To confirm that this would result in the consistent turnover of media in all wells, the bioreactor was filled with water and green dye was perfused via the syringes. Concentration close to the original dye concentration in the syringe was established after 48 h and remained stable [Fig. 2(f)]. Thus, this bioreactor design results in the new media exchanging the old media within 2 days with complete mixing.

### B. Cardiac organoid metabolism, but not function or maturity, is affected by perfusion

Following the validation of the fluid kinetics within the bioreactor, the next experiments were aimed at assessing the feasibility of maintaining hCO culture within this system. The major factors assessed were tissue viability, function, maturation, and metabolism.

**FIG. 3.**
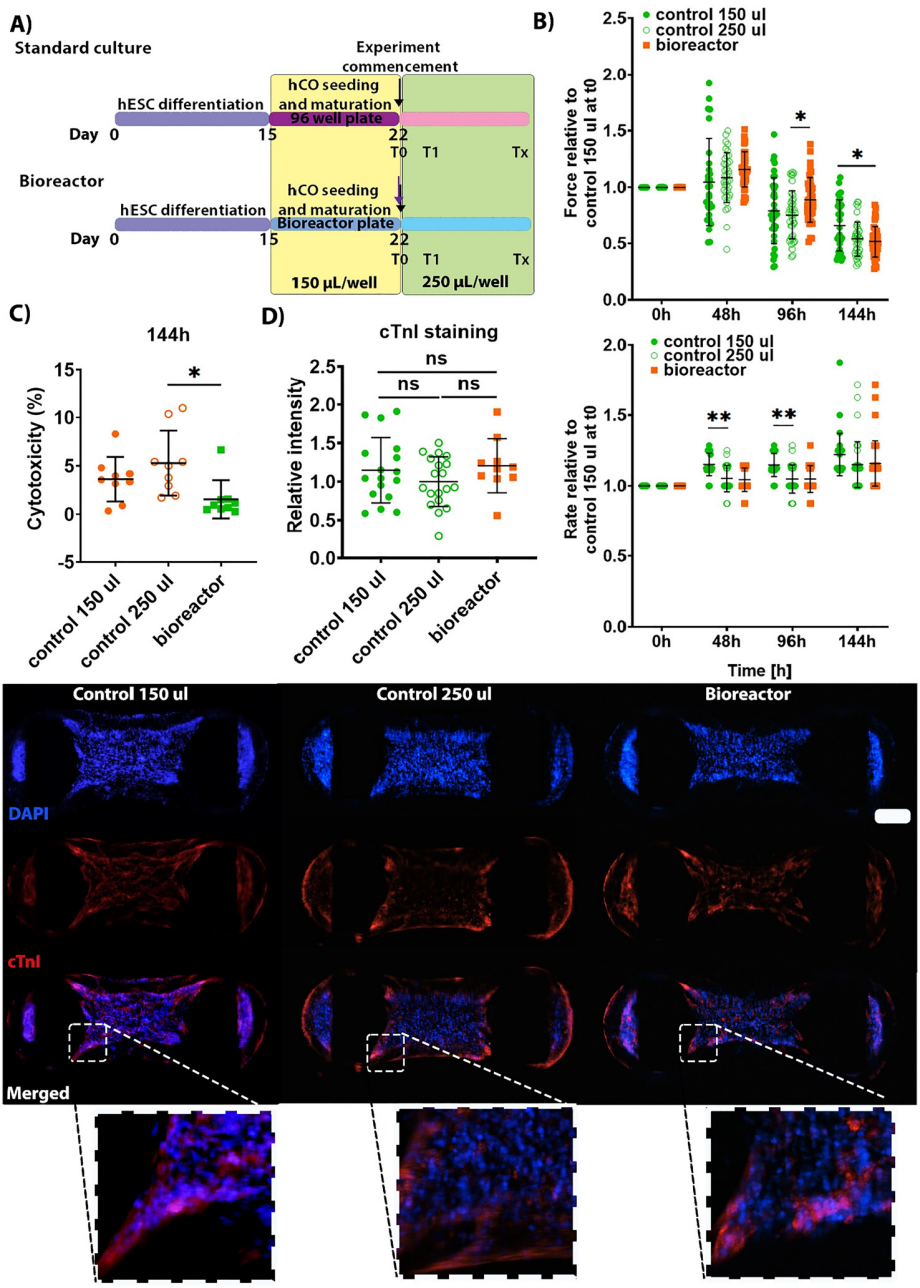
Analysis of hCO culture in the bioreactor. (a) Timeline schematic of standard (control) and bioreactor (perfused) culture. Both were using 150 *μ*l of media per well until “experiment commencement” point, where the medium was increased to 250 *μ*l per well. The medium was increased in standard culture for equivalent comparison. (b) Functional kinetics of the hCO indicating no major differences in contractile force or rate after 6 days. n = 39 hCO across three experiments using HES3 and H9 cell lines. Data presented as mean with SD. ** p < 0.01, *** p < 0.001, and **** p < 0.0001 by two-way ANOVA with Sidak's multiple comparisons test. (c) Cytotoxicity assay measuring the release of LDH into the media shows little cellular death from bioreactor cultured hCO. n = 9 hCO from three separate experiments using HES3 and H9 cell lines. Data presented as percentage of death; the equation used for this calculation can be found in Sec. IV. Data presented as mean ± SD. ** p < 0.01 by two-way ANOVA with Sidak's multiple comparisons test. (d) Maturation marked using cardiac troponin I (cTnI) staining is not altered after 6 days of bioreactor culture. n = 14–29 hCO across three experiments using HES3 and H9 cell lines. Scale bar of 200 *μ*m. Data presented as mean with SD. p > 0.05 was considered insignificant by two-way ANOVA with Sidak's multiple comparisons test.

For both standard (static) and bioreactor (perfused) cultures, the hCO seeding and maturation steps were the same and the static culture was used, but 7 d after seeding, the medium was increased to 250 *μ*l and perfusion began for the bioreactor plate [Fig. 3(a)]. The increase in the medium volume could have an impact on the hCO behavior due to the difference in oxygen availability. Therefore, we used both the regular volume of 150 *μ*l and an increased volume of 250 *μ*l using the standard culturing method as controls for the bioreactor. There were slight functional differences between culturing conditions, but overall, all culture systems exhibited a similar decrease in the force over time and maintenance of the contraction rate [Fig. 3(b) and raw functional data available in Fig. 1(a) in the supplementary material]. Stable kinetics indicate cellular viability over the culture period, which has also been confirmed with an lactate dehydrogenase (LDH) assay at the 144 h time point [Fig. 3(c)]. Furthermore, we assessed cardiac maturity by measuring the abundance of the mature form of cardiac troponin I, type 3 (cTnI),^15^ finding no difference between the cultures [Fig. 3(d)]. Overall, increased medium volume and perfusion does not impact cardiac cell viability, function, or the maintenance of maturity.

### C. Lactate supplementation leads to a steady state in hCO culture

We next assessed the cellular function. First, we evaluated the differential metabolic performance in wells across the plate [Fig. 2(a) in the supplementary material]. The sampled metabolites, glucose, pyruvate, and lactate, and static culture showed little variability due to the position Fig. 2(a) in the supplementary material]. Furthermore, we assessed the hCO function across wells in the case of influence caused by inlet and outlet positioning on the cellular milieu, finding no major differences depending on hCO positioning [Fig. 2(b) in the supplementary material].

**FIG. 4.**
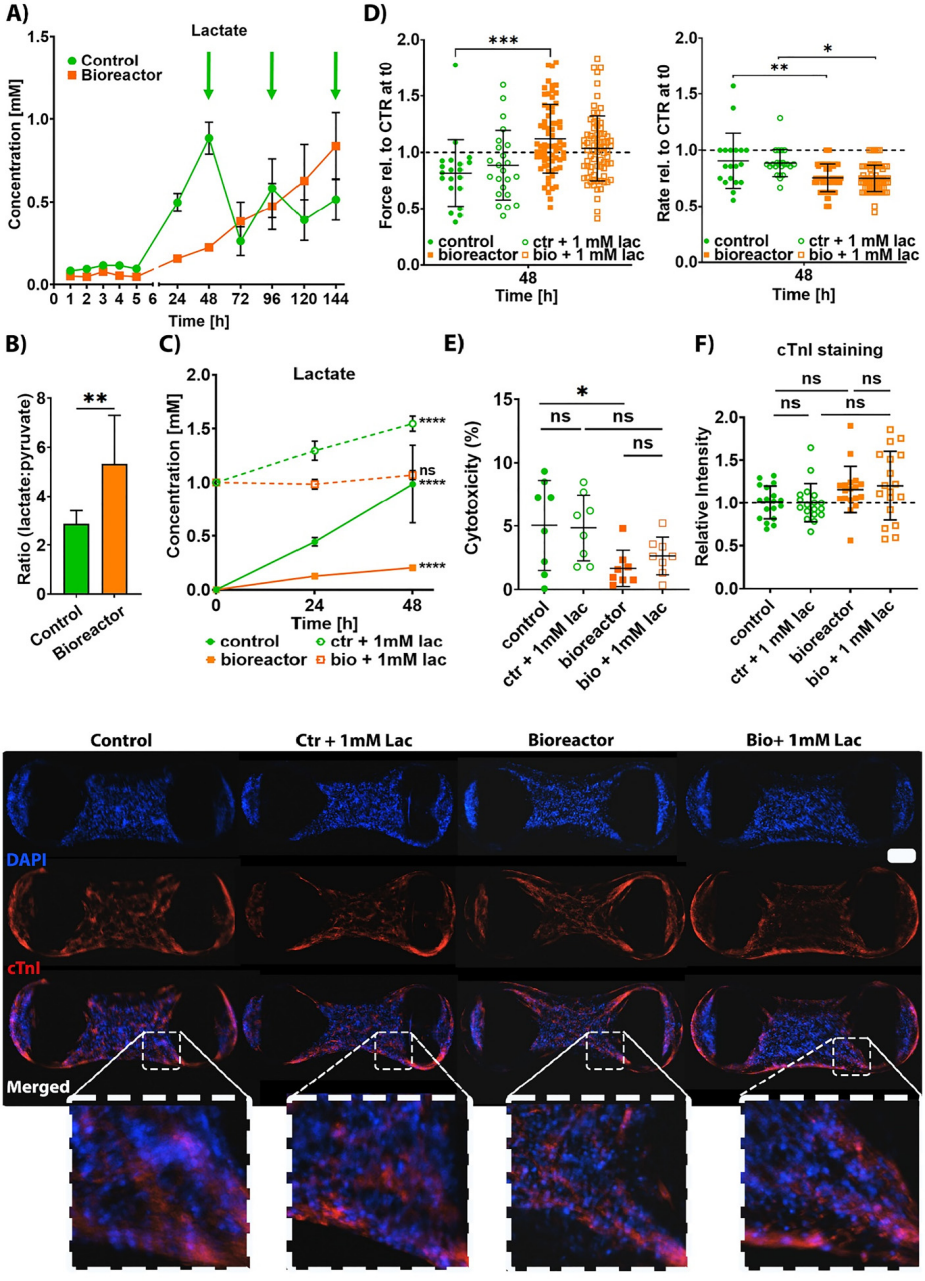
Change in the metabolic environment leads to a stable environment-using bioreactor. (a) Lactate concentration in the medium during culture. Arrows mark the medium changes in control cultures. n = 3 experiments with three sampling places per row each time corresponding to a different well location. Data presented as mean ± SD. (b) Lactate to pyruvate ratio increases double fold in perfused culture as compared to static culture. n = 6 samples across three experiments using HES3 and H9 cell lines. Data presented as mean ± SD of lactate to pyruvate ratio across time. ** p < 0.01 by unpaired t-test. (c) Concentration of lactate in the media reaches a steady state in 1 mM lactate and 0.2 mM pyruvate supplemented bioreactor culture after 48 h. n = 3 experiments with three sampling places each time corresponding to a different well location. Data presented as mean ± SD. p > 0.05 deemed insignificant, **** p < 0.0001 by two-way ANOVA with Sidak's multiple comparisons test. (d) Contractile properties of hCO do not change with the addition of 1 mM lactate and 0.2 mM pyruvate in static or bioreactor culture. n = 23–60 hCO across three experiments using HES3 and H9 cell lines. Data presented as mean ± SD. * p < 0.05, ** p < 0.01, and *** p < 0.001 by two-way ANOVA with Sidak's multiple comparisons test. (e) There is no lactate specific induced cellular cytotoxicity as presented with LDH cytotoxicity assay. N = 8 hCO from three separate experiments using HES3 and H9 cell lines. Data presented as percentage of death; the equation used for this calculation can be found in Sec. IV. Data presented as mean ± SD. p > 0.05 was considered insignificant by two-way ANOVA with Sidak's multiple comparisons test. (f) Maturity marker cTnI was not altered with 1 mM lactate and 0.2 mM pyruvate. n = 6–18 hCO across three experiments using HES3 and H9 cell lines. The scale bar is 200 *μ*m. Data presented as mean ± SD. p > 0.05 by one-way ANOVA with Sidak's multiple comparisons test.

Therefore, we proceeded to examine how well the perfusion system removes “metabolic by-products,” focusing on lactate, which was not present in the naïve culture media. We observed the spikes of lactate in standard culture [green line, Fig 4(a); arrows indicate that the medium changes after sample collection] caused by complete medium exchange, typically performed in cell culture. In the bioreactor with a continuous perfusion rather than medium changes, a stable rise in the total lactate occurred over time (orange line). hCO cultured in a bioreactor for 7 days did not reach a point of lactate equilibrium, indicating continual production under these culture conditions. The highest peak for standard culture was 1 mM, which constitutes a physiological concentration of this metabolite in human plasma.^16^ We also measured medium pyruvate, since the ratio between lactate and pyruvate (L:P) reflects the intracellular redox state.^17^ The L:P ratio rises for both cultures, with a bioreactor culture's ratio being almost twofold higher than a static culture after 144 h [Fig. 4(b)]. This is indicative of a more reduced (higher NADH/NAD+) intracellular environment.

Heart has been shown to have higher utilization of lactate over glucose *in vivo*.^18^ Additionally, elevated levels of circulating lactate have been associated with pathologies, such as ischemic myocardium.^19^ Therefore, lactate has a dual role as an “energy substrate” and a “prognostic biomarker”; however, lactate is not supplied in a standard culture media.

To look at this further, we challenged both culture systems with 1 mM lactate together with 0.2 mM pyruvate (5:1 ratio), which reflected the bioreactor culture at 144 h. This suppressed the net lactate output in both cultures, reducing lactate production by half in the standard culture and leading to the steady state in a bioreactor [Fig. 4(c)]. This indicates that the balance between lactate consumption and production differed between the two systems, being in equilibrium for the bioreactor [Fig. 4(c)]. Despite these metabolic differences, lactate supplementation did not impact hCO function [Fig. 4(d)], viability [Fig. 4(e)], or maturity [Fig. 4(f)].

### D. Lactate addition does not alter hCO transcriptome

**FIG. 5.**
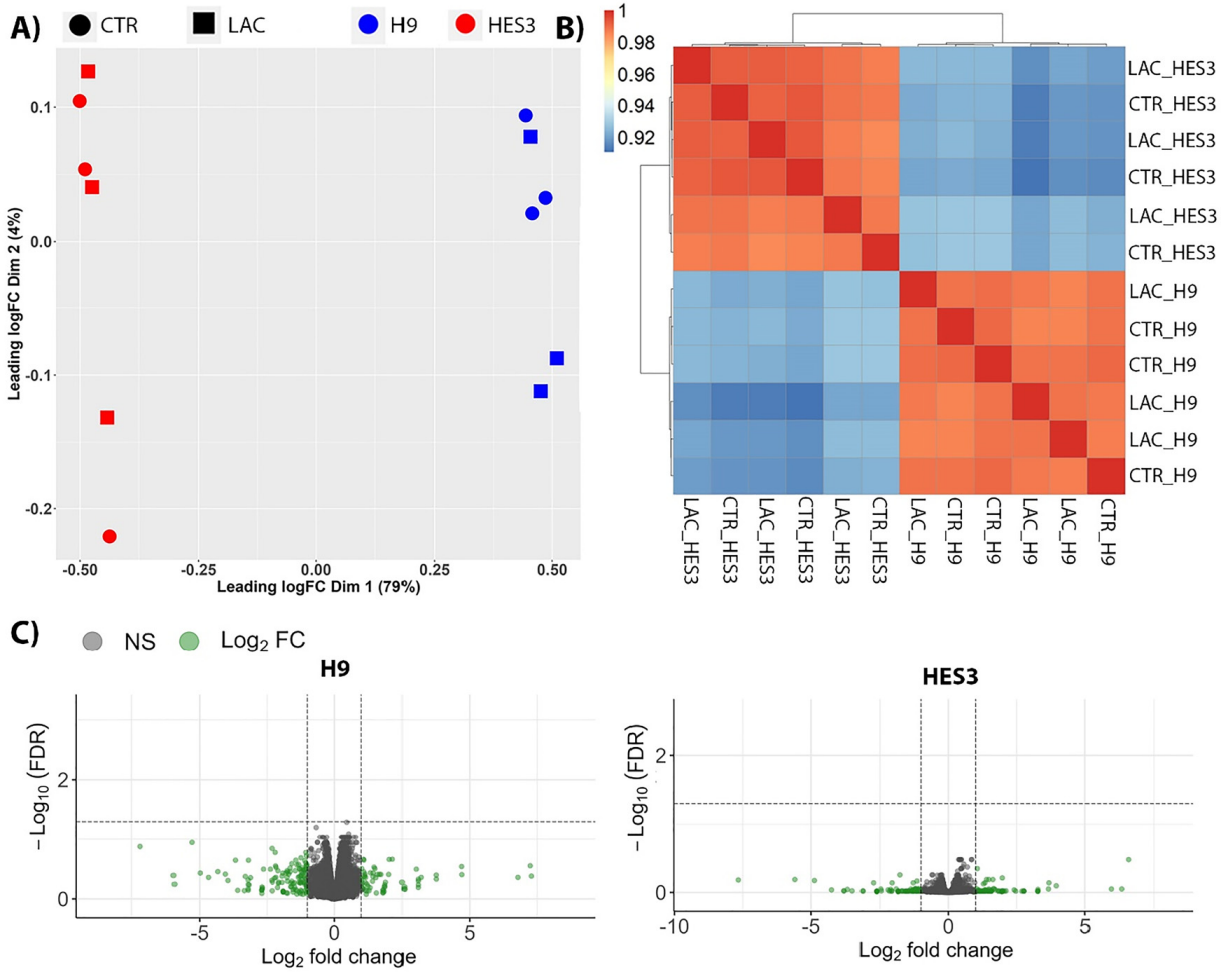
Metabolic conditions do not alter the hCO transcriptome within the bioreactor. (a) MDS plot showing that the variability is between H9 and HES3 cell lines and limited variability between control and 1 mM lactate with 0.2 mM pyruvate treated hCO. n = 2 separate experiments, each with different cell lines, with three independent rows from the bioreactor for each cell line (each point represents pooled 9–10 hCO). (b) Pearson correlation heat map presenting the clear clustering of each experimental group separate to the other and co-clustering of the control and treated samples. The r > 0.92 suggests high quality of data and limited variability. (c) Volcano plots indicating the lack of DEGs in both cell lines between treatment methods, green dots indicate log_2_ FC >1 or <−1.

This implies an adaptive response to changes in the nutrient conditions. To explore this, we evaluated the impact of lactate (with pyruvate) supplementation on the transcriptome using RNA sequencing. Due to preserved functional parameters and cTnI expression between static and bioreactor cultured hCO (Fig. 4), we focused only on the hCO cultured in the perfusion system. Non-equilibrium (no lactate supplementation) vs equilibrium (lactate supplementation) were compared [Fig. 4(d)]. This dataset included hCO generated from two different cell lines, H9 and HES3, the same as the previous experiments. Using multidimensional scaling (MDS) on a full transcriptome dataset, the majority of variation (79%) was accounted for by principal component 1 (PC1), which segregated samples based on cell line [Fig. 5(a)]. There was little segregation between lactate supplementation and their respective control samples. These findings were corroborated in the Pearson correlation heat map with unsupervised hierarchical clustering [Fig. 5(b)].

Due to cell lines segregating separately, we considered each of them individually for further analysis. We found no differentially expressed genes (DEGs) between lactate supplementation and the respective control samples within each cell line [false discovery rate (FDR) <0.05, Fig. 5(c)]. This was despite the very tightly clustered and highly correlated samples within each condition, indicating very limited variability [Figs. 5(a) and 5(b)]. To explore reasons behind the visible segregation between cell lines and not by supplementation, we considered that there may be differences in the representation of cellular populations that would contribute to the seen variation between the experiments. We looked at the expression of specific markers of major cellular populations contributing to the hCO composition, finding that *TNNT2*, *TTN* (cardiomyocyte markers), and *COL1A1* (fibroblast marker) were unchanged across conditions. Markers for epicardial progenitors (*WT1, TBX18*, *TCF21*) and endothelial cells (*PECAM1, CDH5*) differed between cell lines. However, it was not lactate supplementation that altered these cell populations (Fig. 3 in the supplementary material). Together, this indicates minimal changes in transcriptional regulation in response to lactate (with pyruvate) supplementation.

## III. DISCUSSION

Modern cell culture is performed in multi-well plates, typically under static culture conditions that do not provide environmental equilibrium. To overcome this issue, we have developed a simple bioreactor that incorporates perfusion and stirring to rapidly equilibrate the environment. With this setup, we were able to evaluate the impact of culture conditions on hCO, demonstrating that their contractile function and transcriptome are robust in response to environmental fluctuations.

Bioreactors are traditionally used as a scale up of cell culture for industrial scale production,^20–22^ which is not suitable for prototyping and basic research. Typically, research endeavors focus on microscale designs to precisely regulate the conditions while trying to minimize the reagents used.^23,24^ Our bioreactor takes the advantageous aspects such as mixing to improve tissue oxygenation and propagation of nutrients typically important for industrial scale production^25,26^ and combines this with regulated controlled microenvironment for screening more typical of microfluidic devices.^27,28^ At the same time, we focused on minimizing potential disadvantages such as high shear stress caused by stirring^26,29,30^ or incompatibility with molecular techniques, sampling or imaging. There are many platforms used for cardiac organoid culturing mostly relying on regular medium exchanges (for example, Refs. 31–35) with only a few platforms incorporating perfusion.^5–8^ Together, our system has several technical and operational attributes that potentially make it useful to the field: (1) It is the first high throughput bioreactor used to culture hCO. (2) The bioreactor is easy to produce and operate. (3) It is able to keep sterile and functionally intact tissues for at least a week (tested length). (4) It is compatible with PDMS-based organoid culturing platforms as well as molecular and microscopy techniques. (5) As it is an open system, sampling or treatments can be easily applied throughout the duration of an experiment. (6) Each corridor can run a separate condition by the use of different culturing media or additives while maintaining the same flow rate, allowing for simultaneous and comparable experiments within one platform, reducing the cost and labor of experimental series. (7) Different organoid types could potentially be co-cultured in adjacent wells within a corridor, allowing for studies on inter-organ communication.

Having an “open system” for sampling can raise a question of bioreactor repeatability and issues with sterility. For sampling in our bioreactor, the pumping system was stopped, the lid was removed under sterile tissue culture conditions, and the samples were taken. The lid was then replaced, the bioreactor was put back into the incubator, and the pumping system was started again. This did not result in any issues of reproducibility or cross-contamination by spilling between the channels. This robustness can be attributed to the slow flow rate used and quick reestablishment of medium homogeneity by the orbital shaker.

We acknowledge that the need of a pumping system and orbital shaker may impose additional costs and space. In particular, we use two pumps, one to add media and one to remove to keep the medium height stable as this can impact oxygen diffusion if it is altered. Still, those costs and space requirements are lower than the need to use the clean room for fabrication or obtaining a commercial bioreactor system. One of the limitations of our setup is a uniform and singular flow rate across all channels. Optionally, multiple syringe pumps could be used to obtain multiple rates, but this would increase the cost. Self-assembled designs could also be used^36^ but also require additional expertise. There are a few other multiwell-based platforms that also reliant on pumping systems,^5,6^ but those not using pumping systems usually depend on gravitational forces^8^ preventing absolute control over the flow rate and still require the supply and removal of media manually creating intermitted medium changes similar to standard culturing.

We found that perfusion impacts metabolism but not viability, maturity, or contractile function. Other systems have indicated differences in such parameters when comparing perfused systems with static culture.^37–41^ However, most of those studies are performed on cell monolayers, which behave differently to more a complex and biomimetic organoid structure, potentially explaining these differences. Thus, it is important to perform such analyses to ensure that the added complexity of a perfusion system is beneficial to the model being cultured.

By measuring lactate production, we observed several clues that cellular metabolism differed between static and perfusion culture systems. First, the lactate/pyruvate ratio was higher under perfusion [Fig. 4(c)], indicative of a higher cytosolic NADH/NAD^+^ ratio^17^ and, thus, a more reduced intracellular environment. Second, upon lactate supplementation, net lactate efflux ceased in the perfusion cultures but not in the static cultures [Fig. 4(d)]. hCO under perfusion are likely reducing endogenous lactate production while adapting to the presence of the exogenous sources. This suggests either reduced efflux, higher lactate consumption, or lactate production matching the consumption under perfusion. Only a handful of studies measure the redox state of the cultured human cells in the perfused culture.^42,43^ Those studies have indicated a beneficial influence of perfusion on the redox state and mitochondrial function.^43^ Our bioreactor data are consistent with these previous studies, indicating that the redox state was shifted toward a more reduced environment.

The addition of a physiological concentration of lactate to our bioreactor did not change hCO function, maturity, or transcriptome. While there were batch differences reflecting changes in the cellular composition, which could be more consistent with our next generation serum-free protocols,^44^ there was no change caused by lactate (with pyruvate) addition. This shows how resilient the heart is in the face of changing substrate availability under healthy and physiological conditions, likely due to its ability to switch between fuels seamlessly while having to consistently and persistently contract. It would be interesting in the future organoid research to assess the impact of metabolic substrate changes under stressed conditions, including ischemia, inflammation, or nutrient excess, whereby the utilization of different substrates can become more important,^45–54^ and to see whether the transcriptome is affected by metabolic provision under such conditions. Our bioreactor provides an ideal platform for such investigations, as well as the impact of flow rates, shear stress, and tissue–tissue communication in future studies.

## IV. MATERIALS AND METHODS

### A. Maintenance of human pluripotent stem cells

The use of human pluripotent stem cells was approved by QIMR Berghofer's Ethics Committee (P2385) and was carried out according to the National Health and Medical Research Council of Australia (NHMRC) regulations. The hESC lines H9 (female) and HES3 (female) were obtained from WiCell (WA-09, ES03 respectively) where informed consent was obtained from all subjects. hPSC were cultured in mTeSR+ (STEMCELL Technologies, 100-0276) with 0.5% P/S (Gibco, 15140122) on a Matrigel (Corning, 354277) coated flasks. The culturing medium was changed every 2–3 days. ReLeSR (STEMCELL Technologies, 100-0484) was used for the passaging of the cells every 3–4 days and seeded at 16 000 cells/cm^2^. DNA fingerprinting and karyotyping were performed for quality control.

### B. Cardiomyocyte differentiation

Cardiac cells were obtained from human pluripotent stem cells using already established protocols.^9,14^ All cell lines were differentiated using the same protocol. Briefly, RPMI 1640 with GlutaMAX (Gibco, 61870036), 200 *μ*M L-ascorbic acid 2 phosphate sesquimagnesium salt hydrate (ASC) (Sigma-Aldrich, A8960), 1% P/S (RPMI basal media), 2% B27− (Gibco, A1895601) supplemented with 1 *μ*M CHIR-99021 (Stem Cell Technologies, 72054), 5 ng/ml BMP-4 (R&D Systems, 314BP050CF), 9 ng/ml, activin A (R&D Systems, 338AC050), and 5 ng/ml FGF-2 (R&D Systems, 233FB025) was changed daily for 3 days to achieve the differentiation of hPSCs into cardiac mesoderm. This was followed by 3 days of RPMI basal media with 2% B27− supplemented with 5 *μ*M IWP-4 (Stem Cell Technologies, 72554) to obtain hPSC-cardiac differentiation. Subsequently, RPMI basal medium containing 2% B27+ (Gibco, 17504044) with 5 *μ*M IWP-4 was applied for 7 days with medium changes every 2–3 days until day 13. From then, differentiating cells were cultured in RPMI basal media with 2% B27+ until cell dissociation at day 15.

### C. hCO formation and culture

#### 1. Fabrication of Heart-Dyno

Heart-Dyno platform fabrication has been described before.^14^ Briefly, to produce Heart-Dyno inserts, the PDMS (Sylgard 184; Dow Corning, 04019862) was mixed in 1:10 ratio of the curing agent to monomer. After degassing, the mixture was poured over the silicone mold. After the repeated degassing process, PDMS was cured at 65 °C for 35 min. The individual Heart-Dynos inserts were then punched using a 6 mm hole puncher and glued using PDMS into the 96-well plate.

Once the inserts were stably glued, they were sterilized using 70% ethanol for 2 h and 80 min UV exposure time. Before cell seeding inlets were coated with 3% bovine serum albumin (BSA) (Sigma-Aldrich, A9418) to inhibit cell attachment to the bottom of the wells.

#### 2. Seeding of hCO

hCO formation and maintenance have been described before.^14,55^ After 15 days of the differentiation process, cells were harvested using 0.2% collagenase (Sigma-Aldrich, C0130) in 20% fetal bovine serum (FBS) (Gibco, 10099141) in phosphate-buffered saline (PBS) (Gibco, 21600010) for 1 h at 37 °C, followed by suspension in 0.25% trypsin-EDTA (Gibco, 25200072) for 10 min with agitation. The colonies were then filtered through a 100 *μ*m mesh cell strainer (BD Biosciences, 352360) and centrifuged at 300 × *g* for 3 min. Heart-Dyno mixture was prepared by mixing 150.2 *μ*l Collagen I (Devro), 18 *μ*l 10× DMEM (Gibco), 22.5 *μ*l 0.1 M NaOH (ChemSupply), and 33.3 *μ*l Matrigel (Corning), and 6.6 × 10^6^ cells were resuspended in αMEM+++ [MEMα (Gibco, 32561037) with 10% FBS, 1% P/S and 0.2 mM L-ascorbic acid] for a combined volume of 145.6 *μ*l, for 96 hCO. 3.6 *μ*l of Dyno mixture was pipetted per each well. The seeded plate was centrifuged at 100 × *g* for 30 sec and incubated at 37 °C, 5% CO_2_ to gel. After 40 min incubation, 150 *μ*m of αMEM+++ was added per well and changed after 2 days for Maturation Media [MM; DMEM (Gibco, A1443001)] with 1% P/S, 0.2 mM L-ascorbic acid, 1% GlutaMAX (Gibco, 35050061), 4% B27−, 100 *μ*M palmitate (Sigma-Aldrich, P0500), 1 mM Glucose (Sigma-Aldrich, G5767), and 33 ng/ml aprotinin (Sigma-Aldrich, A3428)]. MM was used for 5 days with change every 2–3 days, followed by Weaning Media [WM; DMEM with 1% P/S, 200 mM ASC, 1% GlutaMAX, 4% B27−, 10 *μ*M Palmitate, 5.5 mM glucose, 1 nM insulin (Gibco, 12585014), 33 ng/ml aprotinin] for 7–9 days with medium change every 2–3 days.

### D. Bioreactor fabrication

#### 1. Topper design

Mold for the topper was designed in Tinkercad (AutoDesk) and 3D printed in Eco-ABS filament (Imaginables) using a Dremel 3D45 3D Printer (Core Electronics, CEO6228) using the pre-programmed parameters. PDMS mixture (10:1) was prepared and cast off the mold. Solidified topper was glued using liquid/uncured PDMS (“PDMS glue”), by pouring uncured PDMS onto the 96-well plate using a syringe, to the standard 96-well plate with Heart-Dyno inserts glued at the bottom. To ensure the proper attachment of the topper, a pulling force has been used. To prevent potential leakage between the corridors, each section was filled with dye water one at a time. Any holes were patched using PDMS glue.

#### 2. Optimized final design

Modified 96-well plate construct was designed in TinkerCAD (AutoDesk) and 3D printed in Eco-ABS filament (Imaginables) using the Dremel 3D45 3D Printer (Core Electronics) using the pre-programmed parameters. Bioreactor design can be found in publicly available Autodesk TinkerCAD Gallery under the name “Tissue Bioreactor_96 well format” at https://www.tinkercad.com/things/5×5r1130mKY.

PDMS mixture (10:1) was prepared and applied onto the bottom of the 3D printed part and allowed to degas for 30 min. Then, the Heart-Dyno was aligned manually on the bottom of the 3D print and baked at 65 °C for 1 h.

The standard 96-well plate lid had holes drilled for tubing entrance. SILASTIC tubing (John Morris Scientific, 2415691) was then placed through them on one end and connected to 18G blunt needles (BD, 300204) mounted on Luer Lock containing syringes (TERUMO). Syringes were attached to the syringe pumps (Adelab Scientific, 1600 and NE-1800) set at 62.5 *μ*l/h flow rate according to manufacturer's instructions.

On day 7 from hCO seeding, the well plate culture was maintained as usual, whereas the bioreactor was placed on the oscillating orbital shaker (Thermo Scientific, 88881102) within an incubator, set at 37 °C, 5% CO_2_, and connected to syringe pumps (Adelab Scientific, 1600 and NE-1800), which remained outside of the incubator, providing perfused flow. Media in the syringes was covered with aluminum foil to prevent light exposure.

### E. Bioreactor optimization

#### 1. Shaker equilibration

Bioreactor was placed onto the shaker without tubing connections, 250 *μ*l of water was placed in 11 wells, and the same volume of 1.5625% (v/v with water) green dye (Green: 102 and 133; Queen) was added to the first well. Then, the shaker was turned up to 60 rpm. Samples of 100 *μ*l were collected using multichannel pipet every 20 s and placed in a separate standard 96-well plate. Samples were scanned using a spectrophotometer (BioTek Synergy H4 Hybrid Multi-Mode Microplate Reader) at 425 nm and compared to the standard curve placed on the same plate.

#### 2. Flow rate determination

Syringes were filled with 10 ml of 0.78125% green dye (Queen) and connected to the bioreactor via tubing. Bioreactor was filled with water (250 *μ*l per well), and syringe pumps were activated at 62.5 *μ*l/h flow rate. The bioreactor was stopped during time-points and taken to the spectrophotometer. The plate was scanned at 425 nm with controls of water and green dye dilutions up to 0.78125% at each point. The bioreactor was run until the absorbance in all wells matched the original input dye's absorbance.

### F. Recording and analysis of functional kinetics

Videos of hCOs were captured using a Leica DM6 microscope with a Leica Thunder imager using LAS X software as well as an ANDOR WD Revolution microscope with a BF-Zyla sCMOS camera using Metamorph software. Recorded videos were 10 s time lapses of each hCO contracting at 37 °C, in 5% CO_2_.

Custom batch processing files were written in MATLAB R2018a (Mathworks): (1) converting stacked .tiff image files into .avi movie files; (2) tracking pole deflection (using vision.PointTracker), as approximation for contraction, (3) producing a force over time figure, and (4) exporting batch data into an Excel (Microsoft) spreadsheet. Formulas used to determine contractile force have been described.^14^

### G. Cytotoxicity assay

Cytotoxicity Detection Kit (LDH) (Roche, 11644793001) has been used to assay cellular death using cellular media, according to the manufacturer's instructions. Absorbance was measured using Biotek Synergy H4 Hybrid Multi-Mode Microplate Reader (Agilent). Cytotoxicity was calculated as

$$\text{\%}\;\mathrm{Cytotoxicity}=\frac{\mathrm{Treated}\;\mathrm{LDH}\;\mathrm{activity}-\mathrm{Spontaneous}\;\mathrm{LDH}\;\mathrm{activity}}{\mathrm{Maximum}\;\mathrm{LDH}\;\mathrm{activity}-\mathrm{Spontaneous}\;\mathrm{LDH}\;\mathrm{activity}}\times 100\text{\%}.$$

Maximum LDH activity was measured by crushing a hCO.

### H. Immunohistochemistry

hCO were fixed using 1% paraformaldehyde (PFA) (Sigma-Aldrich, P6148) at room temperature for 1 h, followed by washing with PBS twice. After fixing, hCOs were left overnight in a blocking buffer followed by cTnI (Abcam, ab102331) primary antibody diluted in the blocking buffer and left to oscillate at 45  on a rocker (Stuart^®^ Mini See-Saw Rocker SSM4) overnight at 4 °C. The samples were then washed three times, 5 min each, with blocking buffer. Secondary antibodies, Hoechst (Life Technologies, H3570) and Alexa Fluor 555 (Life Technologies, A-21422), were then applied, diluted in the blocking buffer, and left overnight on a rocker at 4 °C in darkness. hCOs were washed twice with blocking buffer, 5 min each time, and the blocking buffer was then exchanged with PBS. The plate was covered with aluminum foil and stored at 4 °C until imaging.

### I. Relative intensity readings

Whole tissue imaging was performed using the Leica DM6 microscope and Leica Thunder imager with LAS X software. Custom batch processing files were written in MATLAB R2018a (Mathworks) removing background, calculating image intensity, and exporting batch data to an Excel (Microsoft) spreadsheet.

### J. LC-MS

The extraction was performed by using 96 deep well plates, where 8 *μ*l aliquots of medium samples were diluted with 4 vol of water. This was followed by the addition of 160 *μ*l extraction buffer, which consisted of a 1:1 (v/v) mixture of LC-MS grade methanol (Thermo Fisher, FSBA 456-4) and acetonitrile (Thermo Fisher, FSBA955-4) supplemented with the following internal standards: 1 *μ*M D-camphor-10-sulfonic acid (CSA) and 1 *μ*M 2-morpholineethanesulfonic acid (Wako, 037-01032; Sigma-Aldrich, M2933, respectively). The plate was sealed with a silicone mat, and its contents were mixed by inversion and then centrifuged using swing bucket Eppendorf benchtop centrifuge at 1100 × *g* at 4 °C for 20 min. 160 *μ*l of supernatant was transferred to a 1.5 ml Eppendorf tube and lyophilized using a GeneVac EZ2. Samples were then re-suspended in 40 *μ*l ACN:water (1:1) and centrifuged for 20 min at 16 000 × *g* at 4 °C. 30 *μ*l of supernatant was transferred to a high-performance liquid chromatography (HPLC) vial and subjected to LC-MS (HPLC: Agilent 1290 Infinity II, MS: Agilent 6470 QQQ, LC column: Agilent InfinityLab Poroshell 120 HILIC-Z, 2.7 *μ*m, 2.1 × 100 mm^2^, PEEK lined) with a calibration curve prepared in the same way using matrix. Calibration standards (media spiked with known concentrations of lactate and pyruvate) were extracted and analyzed with the experimental samples.

Buffer A was 90:10 (v/v) acetonitrile/water containing 10 mM ammonium acetate and 5 *μ*M medronic acid (Sigma-Aldrich), and buffer B was water containing 10 mM ammonium acetate and 5 *μ*M medronic acid. The medronic acid was included as an additive to improve sensitivity and reduce peak-tailing.^56^ In conjunction, the LC system was deactivated with 0.5% (v/v) phosphoric acid prior to use, according to the manufacturer's protocol. The LC gradient was as follows: 0 min, 10% B, 250 *μ*l/min; 2 min, 10% B, 250 *μ*l/min; 4.5 min, 20% B, 250 *μ*l/min; 4.6 min, 40% B, 250 *μ*l/min; 6.6 min, 40% B, 250 *μ*l/min; 6.9 min, 10% B, 500 *μ*l/min; 10.9 min, 10% B, 500 *μ*l/min; and 11 min, 10% B, 250 *μ*l/min. The autosampler temperature was 4 °C, the column temperature was 30 °C, and the injection volume was 3 *μ*l. MS analysis was performed using an Agilent 6470 QQQ with Jet Stream Technology Ion Source, with the following parameters: gas temperature at 200 °C, gas flow at 11 l/min, nebulizer pressure at 40 psi, sheath gas temperature at 400 °C, sheath gas flow at 12 l/min, capillary voltage at 3000 V for both negative and positive modes, and nozzle voltage at 0 V for the negative mode and 500 V for the positive mode. Multiple-reaction monitoring transitions were calibrated and optimized using metabolite standards, with transition parameters available upon request. Acquisition was performed with a 10 ms dwell time.

LC-MS data were extracted using Skyline (version 20.2.0.343-a7a9e8c4f).^57^ Metabolite peak areas were normalized to the internal standards and converted to absolute quantities using the calibration standards.

### K. RNA-Seq

RNA extraction of pooled up to 10 hCO was performed using RNeasy Micro Kit (Qiagen) according to the manufacturer's instructions. RNA was quantified using the Qubit 4.0 Fluorometer and Qubit RNA High Sensitivity Assay (Invitrogen). The 4200 TapeStation with the high sensitivity RNA ScreenTape kit (Agilent) was used to analyze the integrity of the RNA, giving an RNA Integrity Number (RIN) for each sample. Samples were normalized to <100 ng RNA in 25 *μ*l nuclease free water. Poly(A)-enriched RNA libraries were prepared using the Illumina Stranded mRNA Prep, Ligation (96 samples), and the IDT for Illumina RNA UD Indexes Set B (UDP0177-UDP019) (Integrated DNA Technologies). Libraries were quantified using the Qubit DNA High Sensitivity Assay (Invitrogen). The quality of libraries was assessed using the 4200 TapeStation with the D1000 ScreenTape kit (Agilent). Single-end 75 bp sequencing was conducted on the NextSeq550 (Illumina). All 16 samples, samples from both experiments, were processed for RNA extraction, library preparation, and sequencing in one batch.

Extracted RNA samples and library preparation were of good quality, as shown by the Qubit reading above 1 ng/*μ*l, RIN scores above 7 for each sample, and clear peak readings from TapeStation (Fig. 4 and Table 2 in the supplementary material).

#### 1. Read mapping and quantification

Basecalling and adapter trimming was completed on BaseSpace (Illumina). Data were downloaded and transferred to the QIMR high performance computing system. “FASTQC” (version 0.11.9) was run for quality control. Reads were aligned using “STAR” (version 2.7.9a) to the GRCh38 assembly with the gene, transcript, and exon features of the Ensembl (release 106) gene model. BAM files for each sample were merged using SAMtools (version 1.9). RSEM (version 1.3.1) was used to quantify expression. Duplicate reads were marked using picard MarkDuplicates in the GATK package (version 4.2.4.1). “RNA-SeQC” (version 2.4.2) was used to compute quality control metrics. org.Hs.eg.db (version 3.14.0) was used to annotate gene biotypes. Protein-coding genes were used for further analysis. Differential expression analysis was performed using edgeR (version 3.36.0). The following cutoffs were used to determine DEGs: false discovery rate (FDR) <0.05. The R function plotMDS() was used for multi-dimensional scaling analysis.

### L. Quantification and statistical analysis

All experiments were performed with multiple replicates per condition in multiple experiments to ensure reproducibility. Functional kinetics data presented in Figs. 3(b) and 4(d) were normalized to their own values at time 0 and to the control values, whereas the cTnI intensity presented in Figs. 3(d) and 4(f) was normalized to control, to account for inter-experimental variability. Data are presented as mean with SD unless otherwise stated. The heat map in Fig. 5(c) was created using Morpheus software (https://software.broadinstitute.org/morpheus), and heat maps in Figs. 2(e) and 2(f) were created using GraphPad Prism 9 (GraphPad Software Inc). Statistical analysis was performed using GraphPad Prism 9 using either unpaired t-test, or one-way or two-way analysis of variance (ANOVA) with Dennett's and Sidak's multiple comparison tests to determine the statistical significance, where * p < 0.05, ** p < 0.01, *** p < 0.001, and **** p < 0.0001 were considered significant. Sample numbers, experimental repeats, statistical analysis, and p values are detailed in each figure legend.

## V. CONCLUSIONS

We have created bioreactor compatible with culture in multi-well plates, which is easy to fabricate and use. This bioreactor does not affect the maturation or functional parameters of hCO, even after stabilizing lactate metabolism. This emphasizes the ability of the human heart to readily adapt to changes in metabolic substrates under physiological conditions. Further exploration of different metabolic substrates in our bioreactor could shed light on their utilization in pathological states of different organ models.

## SUPPLEMENTARY MATERIAL

See the supplementary material for additional supporting data for bioreactor function, bioreactor metabolism, and RNA sequencing.

## Data Availability

The data that support the findings of this study are available from the corresponding author upon reasonable request.
